# How Do Arabinoxylan Films Interact with Water and Soil?

**DOI:** 10.3390/foods8060213

**Published:** 2019-06-17

**Authors:** Cassie Anderson, Senay Simsek

**Affiliations:** North Dakota State University, Department of Plant Sciences, Cereal Science Graduate Program, Fargo, ND 6050, USA; Cassie.anderson@ndsu.edu

**Keywords:** arabinoxylan, films, biodegradability, hydrophilicity, food packaging, sustainability

## Abstract

Biodegradable materials made from cereal arabinoxylan could provide an alternative source of packaging to replace current nonbiodegradable plastics. The main purpose of this research was to determine how arabinoxylan (AX) films made from wheat bran (WB) AX, maize bran (MB) AX, and dried distillers grain (DDG) AX made with either glycerol or sorbitol at varying levels (10, 25 or 50%) interacts with soil and water. The biodegradability of all films ranged from 49.4% biodegradable (DDG AX with 10% sorbitol) to 67.7% biodegradable (MB AX with 50% glycerol). In addition, the MB AX films with 25% sorbitol had the lowest moisture content at 9.7%, the MB AX films with 10% glycerol had the highest water solubility at 95.6%, and the MB AX films with 50% glycerol had the highest water vapor transmission rate (WVTR) at 90.8 g h^−1^ m^−2^. Despite these extreme trends in the MB AX films, the WB AX films were the least hydrophilic on average while the DDG AX films were the most hydrophilic on average. The 18 materials developed in this research demonstrate varying affinities for water and biodegradation. These materials can be used for many different packaging materials, based on their unique characteristics.

## 1. Introduction

Wheat (*Triticum aestivum*) and maize (*Zea mays*) are members of the *Gramineae* family and are two of the top three most commonly produced cereal crops [1,2]. The fruit of these cereals is known as a caryopsis and consists mainly of starch, protein, and non-starch polysaccharides [1,3]. The outer portion of a caryopsis is the bran, which makes up 5 to 14% of the caryopsis depending upon the crop [1,4]. The wheat and maize bran is often discarded as a byproduct of milling as it is not desirable for all products (e.g., refined flour). In addition to bran, the dried distillers grain (DDG) is a byproduct of processing maize into ethanol. 

One of the main components in these three byproducts is arabinoxylan (AX). It is the main type of non-starch polysaccharide present in wheat bran (WB), maize bran (MB), and DDG. Arabinoxylan is present in both wheat and maize cell walls, and it is one of the most common non-starch polysaccharides found on earth [5]. It is made up of a backbone of β-1,4 linked xylose units that have O-2 and/or O-3 linked arabinose substituents [5,6,7]. In addition to AX, two other polysaccharides commonly present in AX are glucose and ferulic acid [8,9,10]. Ferulic acid can be substituted on the xylose backbone in the O-3 and/or O-2 locations [6,11,12]. Arabinoxylan cross-linkages are often formed in the presence of ferulic acid because ferulic acid can couple at the O-5 location via an ester linkage.

Arabinoxylan can be extracted from WB, MB, and DDG through a variety of methods including alkaline extraction [13]. This AX can then be used to make films, which are utilized as a prototype of food packaging when testing material properties. Materials used for food packaging must have adequate mechanical properties and extend the shelf-life of the food being stored. Due to this, AX is often combined with a plasticizer, such as glycerol or sorbitol, to increase both flexibility and strength [14,15].

Food packaging has historically been created from synthetic materials including polyolefins, polyamides, and polyesters [16,17]. These materials have been used because of their desirable barrier properties that assist in elongating the shelf life of the food(s) they were packaging. However, the use of synthetic packaging materials, that are not biodegradable or recyclable, has resulted in many ecological problems. When developing food packaging for future use, biodegradability is an important factor to consider. Utilization of AX from WB, MB, and DDG for use as the basis in food packaging materials will increase the overall sustainability of the food packaging industry because these materials are biodegradable [17].

The objectives of this research were to utilize AX of high purity to create film materials, to determine how the films interact with both water and soil (in aerobic biodegradation), and to determine if these interactions are correlated to the physicochemical properties of the films.

## 2. Materials and Methods

### 2.1. Arabinoxylan Preparation and Characterization

The WB, MB, and DDG utilized in this research were provided by the North Dakota State Mill (Grand Forks, ND, USA), Agricor, Inc. (Marion, Indiana), and Tharaldson Ethanol (Casselton, ND, USA), respectively. These materials were commercially available, standard materials produced by each manufacturer. 

The WB AX, MB AX, and DDG AX used as the basis of the films in this research was alkaline extracted from the three starting materials and subsequently purified using a standard method [18]. The moisture content, protein content, ash content, AX content, Arabinose to Xylose (A/X) ratio, M_w_, polydispersity index, and linkages of the three types of AX were also determined using standard methods [19,20,21,22,23].

The AX content and A/X ratio were determined by derivatizing the samples to alditol acetates and analysis with gas chromatography with flame ionization detection [23]. The gas chromatograph used to determine the AX content was a Hewlett Packard 5890 Series II GC system with a flame ionization detector (Agilent Technologies, Incorporated Santa Clara, CA). The column used was a SupelcoSP-2380 fused silica capillary column (30 m × 0.25 mm × 2 µm) (Supelco Bellefonte, PA). The flow pressure was 0.83 MPa, the oven temperature was 100 °C, the flow rate was 0.8 mL min^−1^, the detector temperature was 250 °C, the injector temperature of 230 °C, and the carrier gas was He. The AX content and A/X ratio were calculated by the formulas:

% AX = [((Arabinose + Xylose)*0.88) ÷ (1000*Weight)]*100(1)

A/X ratio = Arabinose ÷ Xylose(2)

To determine the M_w_ and polydispersity index of the AX, a high performance liquid chromatograph (HPLC) with a multi-angle light scattering detector and a refractive index detector were used. The HPLC used was an Agilent 1200 with a Wyatt Dawn Helios-II multi-angle light scattering detector and a refractive index detector (Agilent Technologies, Incorporated Santa Clara, CA, USA). Two columns were used during analysis: A Shodex OHpak guard column and an SB 806-HQ column. The flow rate during analysis was 0.5 mL min^−1^. The Astra 6.0.5 software and a 3rd order Debye plot with the second-order polynomial fit was used for data analysis [24] The proportional change in the refractive index with changes in polymer concentration for AX were assumed to be 0.146 as previously published in research by Dervilly et al. [25].

For AX linkage assessment, the nuclear magnetic resonance spectrometer (NMR) utilized was a 400 MHz spectrometer (Brunker, Billerica, MA, USA) (Bruker AV3 HD 400 MHZ NMR that had a 5 mm PABBO BB/19F-!H/D Z-GRD Z probe). TopSpin 3.2 software (TopSpin, Billerica, MA, USA) was used to analyze all data [26].

### 2.2. Preparation of Arabinoxylan Films

All films were created according to the methods of Anderson and Simsek [18]. The sorbitol (BioUltra Grade) and glycerol (ACS Reagent Grade) were purchased from Sigma-Aldrich (Saint Louis, MO, USA). Film solutions were made by preparing a 26.7 g kg^−1^ solution of AX in deionized water. The solutions were stirred for 24 h then divided into aliquots and heated at 90 °C for 15 min. Then, sorbitol or glycerol was added (100, 250 or 500 g kg^−1^) and the samples were vortexed. After heating at 90 °C for an additional 15 min, the samples were cast onto polystyrene Petri dishes and dried at 60 °C for 8 h. The films were allowed to finish drying overnight at 23 °C. All films were stored in a Dry-Keeper (Sanplatec Corporation, Catalog No. H42056-0001, Osaka, Japan) with Boveda 49% relative humidity packs (Item No. B49-60-48). Prior to the analysis of the interactions of the AX films with water, all films were conditioned at 23 °C and 50% relative humidity for at least 48 h prior to analyses.

### 2.3. Moisture Content Determination

The moisture content of each film was determined in triplicate following the method of Garcia et al. [27]. To begin, the initial masses of three films from each treatment were determined and recorded. Next, all films were placed into a 110 °C oven for 24 h. After 24 h, the final mass of each film was determined. The moisture content of each film was determined using the following equation:

Film moisture content = [(Initial film mass − Final film mass) ÷ (Initial film mass)]*100 (3)

### 2.4. Water Solubility Analysis.

The percentage of water-soluble material in each film was determined in duplicate following a modified method of Garcia et al. [27]. To begin, each film was carefully cut into a 2 cm × 3 cm piece and the initial mass was recorded. Next, each film was placed into 80 mL of distilled water in a capped glass jar. The jars were then placed onto a Lab-Linee Orbit Shaker (Model No. 3520) and shaken at 75 rpm for one h at 25 °C. After one h, each sample was filtered through an Endecotts Limited (London, England) Steel Mesh No. 325 (Aperture: 45 microns). The solid material remaining was dried at 100 °C for 10 h and the final mass of the material was determined. The percentage of water-soluble material was determined using the following equation:

Water soluble material = [(Initial film mass − Final film mass) ÷ (Initial film mass)]*100(4)

### 2.5. Contact Angle and Wetting Tension Determination

The contact angle and wetting tension of all treatments was determined by following the American Society for Testing and Materials official method D7334-08 in duplicate for distilled water [28]. A Dynamic Contact Angle Analyzer by First Ten Angstroms 125 (Serial No. 980806, First Ten Angstroms, Portsmouth, VA, USA) with a charge coupled device camera was used for testing and calculation of contact angle and wetting tension. A drop of distilled water was applied to the film with a syringe and the contact angle of the drop of water with the film was measured using the dynamic contact angle analyzer instrument [28].

### 2.6. Water Vapor Permeability Analysis

The (WVTR) and permeance are part of the water vapor permeability measurement that was determined according to the American Society for Testing and Materials official method E96/E96M-15 in triplicate [29]. The average thickness of the films was 0.0792 mm. The water method was used, and the relative humidity and temperature were recorded every 30 min. During testing, the mass of each sample was determined using a Mettler Toledo New Classic MF analytical balance (Model No. ML203E/03). The test assembly was as follows: A polystyrene dish (100 mm in diameter) with 20 mL distilled water, two steel metal plates with inner diameters of 5.4 cm and outer diameters of 11.43 cm with plain finishes (Grainger Item No. 22UE14; Model No. U38402.200.0001) were used to hold the specimen flat, and parafilm wax was used to secure the specimen and seal the entire apparatus. The film specimens were 13 mm above the surface of the water. The test was conducted at room temperature, which was 22–25 °C, at the time of the test. The following equations were used to determine the water vapor transmission rate and water vapor permeance, respectively.

Water vapor transmission rate = G ÷ *t*A (5)

where G = mass change (in grams), *t* = time in h, G *t*^−1^ = slope of a straight line in grams per h, A = test area in square meters.

Water vapor permeance = WVTR ÷ Δp = WVTR ÷ [S(R_1_ − R_2_)] (6)

where WVTR = water vapor transmission rate, Δp = vapor pressure difference in mm Hg, S = saturation vapor pressure at test temperature in mm Hg, R_1_ = relative humidity at the source, R_2_ = relative humidity at the vapor sink. In this study R_1_ = 1.0 and R_2_ = 0.26625.

### 2.7. Biodegradability Analysis

The C content of all films was determined using a Primacs TOC Analyzer (Model CS22). The biodegradability analysis of the AX films was completed in triplicate according to a combination of the method of Colussi et al. [30] and the American Society for Testing and Materials official method D5988-12 [31]. Soil collected from Foster County, ND (100 g) was mixed with enough water to reach 60% of the moisture holding capacity (MHC) of the soil (60% MHC = 36.67g/100g soil), and half of the wetted soil was added to a 1-l airtight glass jar. Then, a 400 mg sample of AX film was placed on top of the soil and the film was covered with the remaining soil. A polystyrene cup (50 mL) with 20 mL of 1M NaOH was placed on top of the soil and the jar was sealed tightly. A control sample of soil only was used as a blank, and all samples were stored at room temperature (23 to 26 °C). The CO_2_ production was measured over 145 days. The cups containing NaOH were removed from the jars on days 19, 40, 55, 82, 103, 119 and 145 and replaced with new cups containing fresh NaOH solution. The NaOH was titrated to determine the CO_2_ produced. This titration was conducted by transferring the NaOH that had been stored with each film sample to a flask and adding 5 mL of BaCl_2_ (25%). Three drops of 0.1% phenolphthalein were added and 1M HCl was used to titrate the solution until it turned from pink to white. The following equations were utilized to determine the total amount of biodegradable material in each type of film.

Release of C per 100 g CO_2_ = (1 ÷ mg CO_2_) − (AB)*(Acid molarity)*(Eq.g C-CO_2_) (7)

where A is the amount of HCl spent in reagent blank in mL, B is the amount of hydrochloric acid spent in the sample in mL, acid molarity is the molarity of the HCl in M, and Eq.g. C-CO_2_ is the equivalent gram C-CO_2_.

Percentage of film biodegraded = [(CO_2_ soil with film − CO_2_ soil without film) ÷ (mg C in film)]*100 (8)

The soil (silt loam: 25.8% sand, 57.8% silt, 16.4% clay) used for testing was collected from Foster County, ND June of 2015. The sampling depth of the soil collected was 0 to 15 cm. It was stored at 23 °C in a closed container and handled with gloved hands and clean tools. The particle size was of the soil was less than 2 mm. The moisture holding capacity of the soil was 36.67 g water per 100 g soil. The C:N of the soil was 11:1, and the pH was 6.2.

### 2.8. Statistical Analysis

The experiment utilized a completely random design with a factorial arrangement that was utilized with three factors (AX type, plasticizer type, and plasticizer level). Statistical Analysis Software version 9.3 was utilized for all data analysis [32]. All data was analyzed using ANOVA and Fischer’s protected least significant difference (LSD). The Pearson’s correlation coefficient was utilized to assess the relationships between the five film properties and the physicochemical properties of the films.

## 3. Results and Discussion

### 3.1. Arabinoxylan Characterization

The AX extracts utilized in this research were of a purity of at least 58% as shown in Table 1. In addition, the arabinose to xylose ratio for all extracts was 0.51. The MB AX had the highest M_w_ indicating that this extract had the largest AX polymers, whereas the DDG AX had the lowest M_w_ and polydispersity index indicating these AX polymers were the smallest and the most similar in M_w_. The WB AX had the highest polydispersity index indicating the largest range for AX M_w_ out of the three extracts.

**Figure 1:**
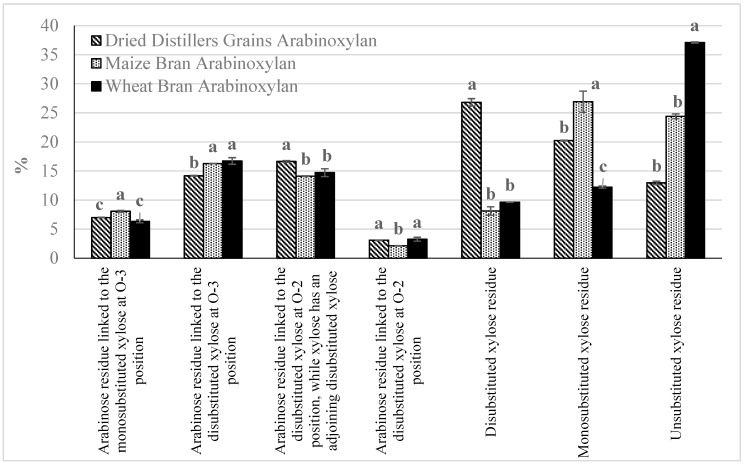
Abundance of seven types of anomeric protons present in dried distillers grain arabinoxylan, maize bran arabinoxylan, and wheat bran arabinoxylan as determined by ^1^H Nuclear Magnetic Resonance Spectrometry. Columns with the same letter for the same proton are not significantly (*p* < 0.05) different, error bars represent standard deviation.

Figure 1 provides the abundance of each type of linkage present in the three AX extracts. The linkage analysis showed that there were significant (*p* < 0.05) variations in the structures of the three types extracted AX. The variation in the three types of AX extracts is most likely due to the differences in the starting material AX structure [33]. These differences in linkages influenced the interactions of films made from these three types of AX with both water and soil in aerobic biodegradation.

### 3.2. Interactions of Arabinoxylan Films with Water

Each of the 18 materials developed in this research interacted with water in different ways as shown in Table 2 and Table 3. The moisture content, water solubility, hydrophilicity (as determined by contact angle), and water vapor permeability are all vital characteristics to food packaging materials. These properties must be carefully characterized so that a proper material can be developed to store food. When the proper packaging material is utilized, it can extend the shelf life of food, which may decrease food waste and increase sustainability in the food industry.

When the type of AX (WB, MB, or DDG) is considered, there are clear trends in the interaction of these AX films with water. Taking all four measurements of the interactions of these 18 types of AX films with water into account, the films made with WB AX appear to be the least hydrophilic and the DDG AX films appear to be the most hydrophilic. First, there were significant (*p* < 0.05) differences in the moisture content of the films. The films with the lowest moisture content were the MB AX films (average moisture content of 13.7%), while DDG AX films had the highest moisture contents (average moisture content of 15.6%). There were also significant (*p* < 0.05) increases in moisture content for films with increasing levels of glycerol. There was a significant (*p* < 0.05) correlation between the polydispersity index of the AX in the films and their moisture content for films made with glycerol (*r* = 0.77). 

As these films became more heterogeneous in M_w_ they became more susceptible to moisture penetration. Second, the DDG AX films had the highest amount of water-soluble material (averaging 85.1%), followed by the MB AX films (averaging 79.1%), and lastly the WB AX films (averaging 39.5%). There were significant (*p* < 0.05) decreases in water solubility when films were prepared with higher levels of the sorbitol or glycerol plasticizers. There was a significant (*p* < 0.01) correlation between the M_w_ of the films and their water solubility (*r* = −0.78 for films made with sorbitol and *r* = −0.91 for films made with glycerol). In addition, there were significant (*p* < 0.05) correlations between an increase in the presence of disubstituted xylose and an increase in the amount of water-soluble material present in the film correlations (*r* = 0.76 for films made with sorbitol and *r* = 0.67 for films made with glycerol). This was due to a decrease in inter-polymer interaction that allowed water to enter and dissolve the material. Third, the WB AX films were the least hydrophilic with average contact angles of 79° and wetting tensions of 13.8 mN m^−1^. The MB AX films were the next hydrophilic with average contact angles of 72° and average wetting tensions of 21.9 mN m^−1^ on average. In general, films made with sorbitol had significantly (*p* < 0.05) lower contact angle than films made with sorbitol, but films made with sorbitol tended to have significantly (*p* < 0.05) higher wetting tension than those prepared with glycerol. The average contact angles and average wetting tensions of the DDG AX films were 55° and 41.4 mN m^−1^, respectively. There was a significant (*p* < 0.01) correlation between the presence of unsubstituted xylose on the AX polymer used in the films and the contact angle of the films with water (*r* = 0.81 for films made with sorbitol and *r* = 0.74 for films made with glycerol) demonstrating that as the branching on the AX polymer increases, the films became more hydrophilic. Fourth, there were significant (*p* < 0.05) differences in WVTR and water permeance among the films. The MB AX films were the most permeable to water vapor with an average WVTR of 64.6 g h^−1^m^−2^ and average permeance of 411.0 g/s·m^2^·Pa. The films made with WB AX were the least permeable to water vapor and had an average WVTR of 54.7 g h^−1^m^−2^ and average permeance of 348.7 g/s·m^2^·Pa. The level of sorbitol did not significantly (*p* < 0.05) affect the WVTR for films prepared from WB AX or MB AX, but the WVTR was significantly (*p* < 0.05) higher in DDG AX films prepared with 50% sorbitol than the DDG AX films with 10% or 25% sorbitol. For the most part, films made with glycerol had significantly (*p* < 0.05) higher WVTR and water permeance than films made with sorbitol.

The interaction of each type of AX film with water depended not only on the type of AX but also on the type of plasticizer present in the film. Glycerol is more hydrophilic than sorbitol and has a greater plasticizing ability [34]. The films developed in this research that were made with glycerol had an average moisture content of 18.9%, while those made with sorbitol had an average moisture content of 11%. This trend was also noted in previous work published by Antoniou et al. [35]. In addition, films made with sorbitol were significantly (*p* < 0.01) more water soluble (with an average of 72.2% water-soluble material) than those made with glycerol (with an average of 63.7% water-soluble material). This result is the same as that found in films made with varying levels of sorbitol or glycerol by Müller et al. [34]. Similarly, films made with glycerol had contact angles of 73° and wetting tensions of 20.0 mN m^−1^ on average, whereas films made with sorbitol had contact angles of 64° and wetting tensions of 31.4 mN m^−1^ on average. Lastly, the films made with sorbitol had a significantly (*p* < 0.01) lower WVTR than those made with glycerol. The films made with sorbitol had an average WVTR of 49.9 g h^−1^m^−2^ and average permeance of 317.4 g/s·m^2^·Pa. The films made with glycerol had an average WVTR of 67.9 g h^−1^m^−2^ and average permeance of 432.4 g/s·m^2^·Pa. Zhang and Whistler also observed this trend in the WVTR of their MB AX films [15].

The amount of plasticizer also played a major role in the interactions of these AX films with water. The moisture contents of the films increased significantly (*p* < 0.01) from 11.4 to 20.0% as the level of plasticizer increased from 10 to 50%. However, there was a decrease in water-soluble material present in the films as the plasticizer level increased. The films made with 10% plasticizer had 75.4% water-soluble material on average, whereas those made with 25% plasticizer or 50% plasticizer had 66.1% water-soluble material or 62.3% water-soluble material, respectively. This trend was also identified by Nazan Turhan and Şahbaz in the films they created [36]. This is most likely due to the increase in intermolecular interactions due to the presence of the plasticizer, which limits the interaction of the AX polymers with water. In addition, as the level of plasticizer increased, there was a significant (*p* < 0.05) decrease in the hydrophilicity of the films as measured by contact angle and wetting tension. As the plasticizer content increased from 10 to 50%, the contact angles of the films increased (on average) from 66° to 71°, respectively. Similarly, the wetting tensions of the same films decreased (on average) from 29 to 24 mN m^−1^ as the plasticizer content increased from 10 to 50%, respectively. These results show the same trend as those of previously published work with variation in plasticizer levels and surface hydrophilicity [37]. Finally, as the plasticizer level in the films increased from 10 to 25 to 50%, the average WVTR increased from 52.0 to 58.2 to 66.5 g h^−1^m^−2^, respectively. In addition, the average permeance also increased from 331.1 to 370.2 to 423.5 g/s·m^2^·Pa as the plasticizer level in the films increased from 10 to 25 to 50%, respectively. This is the same trend observed by Nazan Turhan and Şahbaz in their research on water vapor permeability of films at varying plasticizer levels [36]. These increases in water vapor permeability could be due to the decreased hydrophilicity of the films and the decreased order of the AX polymers allowing more water vapor to pass through.

### 3.3. Arabinoxylan Film Biodegradability

**Figure 2:**
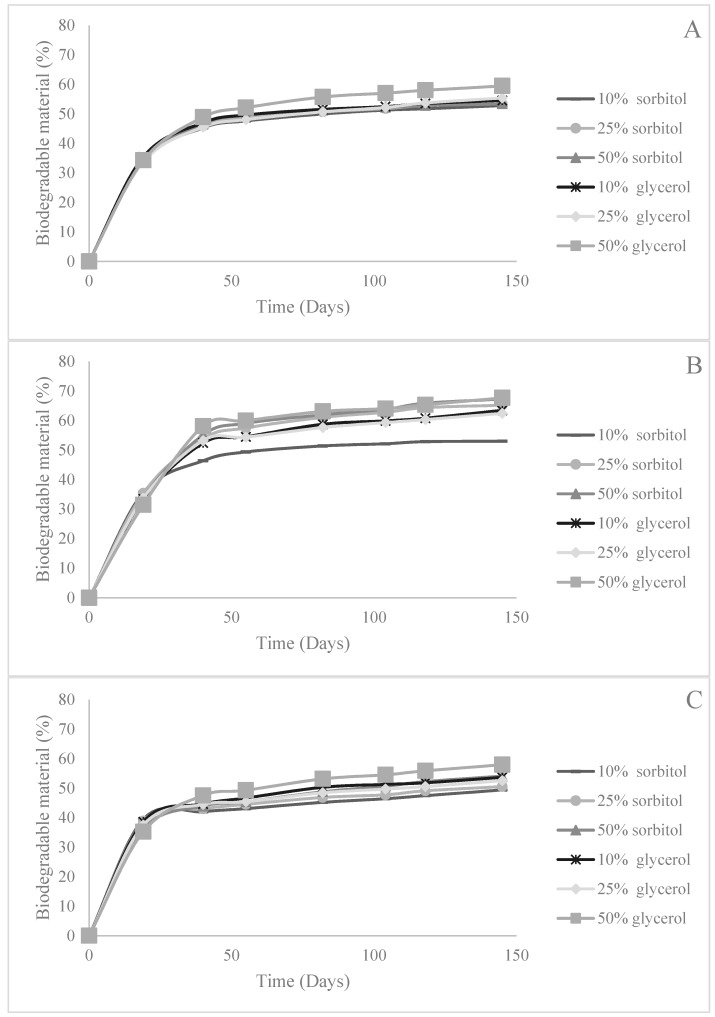
Biodegradability profiles for films made with wheat bran arabinoxylan (**A**), maize bran arabinoxylan (**B**), and dried distillers grain arabinoxylan (**C**).

While food packaging must be strong enough to protect the food it contains, it is also beneficial if it is biodegradable. The biodegradation of bio-based materials such as AX films will vary from material to material, but the microorganisms in soil capable of breaking down AX are those that can produce hemicellulases such as *Bacillus* spp. [38,39]. The biodegradability of the materials developed in this research is given in Table 4. All 18 types of AX film rapidly biodegraded within the first 45 days of measurement followed by a plateau in biodegradation (Figure 2).

It has been documented that during this time, the components of plant-based materials are broken down sequentially [40]. Soluble carbohydrates are broken down first followed by proteins and structural carbohydrates. The hemicellulose and cellulose present are broken down last [40]. The biodegradability profiles for the AX films are given in Figure 2.

On average, the total amount of biodegradable material in each type of films was as follows: 53% in DDG AX films, 55% in WB AX films, and 63% in MB AX films. There was a significant (*p* < 0.01) correlation between the polydispersity index of the films and their biodegradability (*r* = −0.70 for films made with sorbitol and *r* = −0.72 for films made with glycerol). This is indicative of a decrease in biodegradability, as the AX polymers were increasingly heterogeneous in the films, which could have impeded the microbial breakdown of the films. 

In addition, the utilization of glycerol instead of sorbitol increased the total biodegradability of the film by about 3%. Furthermore, when comparing the biodegradability of these films, the general trend was that as the plasticizer level increased so did their biodegradability. These trends in the effects of plasticizer type and level on the total amount of biodegradable material in the AX films are most likely due to the loss of order in the polymers of the films. This loss of order creates a material that can be more easily broken down by the microbes in the soil.

## 4. Conclusions

Biodegradable packaging material must have a balance between being easily degraded after use and having the proper mechanical properties for the particular application of interest. The materials produced in this study show promise as the basis for biodegradable food packaging materials in the future as demonstrated by their mechanical properties and interactions with water. The general trend in water solubility was as follows for these AX films: WB < MB < DDG. In addition, the DDG AX films were the least biodegradable and the MB AX films were the most biodegradable. These trends in the interactions of these films were related to their physicochemical properties including AX M_w_ and AX polydispersity index. While there were fewer clear-cut trends between the physicochemical properties of the films and their biodegradability, all films were at least 49% biodegradable. In addition, as the amount of glycerol or sorbitol in the films increased, the films became less hydrophilic but more biodegradable. The combination of these pieces of information can be utilized to tailor biodegradable packaging materials for food products to ensure maximum shelf life. Overall, each type of film tested in this paper can lend itself to various packaging and materials applications. Some of these films may be better suited to plastic wrapping material, while others would be better to use for plastic bags. It would be greatly beneficial to continue researching the properties of AX-based materials to determine how they would behave under all types of environments including anaerobic biodegradation.

## Figures and Tables

**Table 1:** Proximate compositions (dry weight basis) of alkaline extracted wheat bran arabinoxylan, maize bran arabinoxylan, and dried distillers grain arabinoxylan.

	Moisture (%)	Ash (%)	Protein (%)	Arabinoxylan (%)	Molecular Weight (g/mol)	Polydispersity index
Wheat bran arabinoxylan	12.62	8.55	13.62	72.94	7.12 × 10^6^	1.59
Maize bran arabinoxylan	11.11	1.22	3.89	84.71	7.70 × 10^6^	1.27
Dried distillers grain arabinoxylan	6.31	1.89	14.95	58.05	5.90 × 10^6^	1.04
LSD (*p* < 0.05)	1.39	0.06	1.31	2.97	0.60 × 10^5^	0.23
LSD (*p* < 0.01)	2.56	0.11	2.40	5.45	0.80 × 10^5^	0.31

LSD: Least Significant Difference.

**Table 2:** Interactions of wheat bran arabinoxylan, maize bran arabinoxylan, and dried distillers grain arabinoxylan films with water.

Film Composition	Moisture Content	Water Soluble Material	Contact Angle	Wetting Tension
	(%)	(%)	(°)	(mN m^−1^)
WB AX ^a^ + 10% sorbitol	11.0 ± 1.9	54.7 ± 0.4	62.81 ± 0.66	33.27 ± 0.75
WB AX + 25% sorbitol	11.1 ± 0.3	36.0 ± 2.3	63.97 ± 1.68	31.94 ± 1.91
WB AX + 50% sorbitol	11.6 ± 0.2	44.0 ± 3.3	66.59 ± 0.34	30.02 ± 1.15
WB AX + 10% glycerol	12.1 ± 0.8	38.0 ± 4.7	76.71 ± 0.68	17.02 ± 1.24
WB AX + 25% glycerol	16.0 ± 0.5	30.5 ± 0.1	101.30 ± 0.47	−14.26 ± 0.58
WB AX + 50% glycerol	29.0 ± 0.7	34.2 ± 1.8	102.01 ± 0.39	−15.15 ± 0.49
MB AX ^b^ + 10% sorbitol	11.0 ± 0.3	87.9 ± 0.3	71.03 ± 0.78	23.66 ± 0.93
MB AX + 25% sorbitol	9.7 ± 0.1	86.3 ± 6.6	71.52 ± 0.69	23.08 ± 0.84
MB AX + 50% sorbitol	10.2 ± 0.3	70.5 ± 6.7	74.25 ± 1.04	19.77 ± 1.27
MB AX + 10% glycerol	10.3 ± 0.6	95.6 ± 3.0	71.10 ± 0.66	23.58 ± 0.79
MB AX + 25% glycerol	19.3 ± 0.9	71.7 ± 1.4	82.25 ± 3.00	9.81 ± 3.77
MB AX + 50% glycerol	21.3 ± 1.4	62.5 ± 5.5	64.45 ± 0.51	31.40 ± 0.58
DDG AX ^c^ + 10% sorbitol	10.5 ± 0.5	93.7 ± 2.1	54.23 ± 2.67	42.54 ± 2.75
DDG AX + 25% sorbitol	10.2 ± 1.3	87.3 ± 1.1	52.49 ± 0.01	44.33 ± 0.01
DDG AX + 50% sorbitol	10.6 ± 0.4	89.3 ± 3.2	62.29 ± 0.75	33.85 ± 0.85
DDG AX + 10% glycerol	13.4 ± 0.8	82.6 ± 0.8	61.27 ± 3.56	34.96 ± 3.97
DDG AX + 25% glycerol	11.9 ± 0.1	84.5 ± 5.9	46.67 ± 1.21	49.96 ± 1.12
DDG AX + 50% glycerol	37.1 ± 0.7	73.2 ± 6.6	53.88 ± 0.61	42.91 ± 0.62
LSD (*p* < 0.05)	1.3	8.1	3.06	3.56
LSD (*p* < 0.01)	1.8	11.1	4.20	4.88

Results are expressed as means ± standard deviation. ^a^ Wheat bran arabinoxylan; ^b^ maize bran arabinoxylan; ^c^ dried distillers grain arabinoxylan. AX: arabinoxylan; WB: wheat bran; MB: maize bran; DDG: dried distillers grain.

**Table 3:** Water vapor transmission rate and water permeance of wheat bran arabinoxylan, maize bran arabinoxylan, and dried distillers grain arabinoxylan films.

Film Composition	Water Vapor Transmission Rate	Water Permeance
	(g h^−1^ m^−2^)	(g/s·m^2^·Pa)
WB AX ^a^ + 10% sorbitol	44.8 ± 3.1	285.4 ± 19.5
WB AX + 25% sorbitol	45.4 ± 0.9	289.6 ± 5.6
WB AX + 50% sorbitol	47.3 ± 1.8	301.6 ± 11.8
WB AX + 10% glycerol	56.6 ± 3.8	360.4 ± 24.4
WB AX + 25% glycerol	60.6 ± 7.0	386.0 ± 44.6
WB AX + 50% glycerol	73.7 ± 3.6	469.4 ± 22.6
MB AX ^b^ + 10% sorbitol	50.6 ± 1.8	321.5 ± 11.8
MB AX + 25% sorbitol	53.2 ± 2.9	338.3 ± 18.5
MB AX + 50% sorbitol	54.1 ± 0.8	344.0 ± 5.0
MB AX + 10% glycerol	60.1 ± 1.1	382.4 ± 6.9
MB AX + 25% glycerol	78.9 ± 3.6	502.0 ± 23.2
MB AX + 50% glycerol	90.8 ± 1.6	577.8 ± 10.3
DDG AX ^c^ + 10% sorbitol	48.5 ± 3.1	308.8 ± 19.5
DDG AX + 25% sorbitol	49.8 ± 0.8	316.8 ± 5.0
DDG AX+ 50% sorbitol	55.16 ± 4.45	350.8 ± 28.3
DDG AX + 10% glycerol	51.52 ± 1.60	328.2 ± 10.2
DDG AX + 25% glycerol	60.95 ± 2.72	388.3 ± 17.3
DDG AX + 50% glycerol	78.04 ± 2.46	497.2 ± 15.7
LSD (*p* < 0.05)	5.02	32.0
LSD (*p* < 0.01)	6.73	42.9

Results are expressed as means ± standard deviation. ^a^ Wheat bran arabinoxylan; ^b^ maize bran arabinoxylan; ^c^ dried distillers grain arabinoxylan.

**Table 4:** Carbon contents and total biodegradable material of arabinoxylan films made with arabinoxylan extracted from wheat bran, maize bran, or dried distillers grain.

Film Composition	Carbon Content ^a^	Total Biodegradable Material
	(mg)	(%)
WB AX ^b^ + 10% sorbitol	140.6	52.8 ± 0.59
WB AX + 25% sorbitol	140.6	53.4 ± 1.73
WB AX + 50% sorbitol	141.5	53.4 ± 0.63
WB AX + 10% glycerol	141.4	54.5 ± 0.43
WB AX + 25% glycerol	142.2	55.4 ± 0.34
WB AX + 50% glycerol	135.8	59.5 ± 0.54
MB AX ^c^ + 10% sorbitol	154.3	53.0 ± 0.40
MB AX + 25% sorbitol	152.1	65.2 ± 0.77
MB AX + 50% sorbitol	145.6	67.3 ± 0.07
MB AX + 10% glycerol	148.3	63.4 ± 1.99
MB AX + 25% glycerol	146.6	62.4 ± 1.25
MB AX + 50% glycerol	134.7	67.7 ± 1.41
DDG AX ^d^ + 10% sorbitol	161.3	49.4 ± 0.42
DDG AX + 25% sorbitol	159.4	50.5 ± 0.22
DDG AX + 50% sorbitol	157.4	54.1 ± 0.82
DDG AX + 10% glycerol	153.9	53.7 ± 0.29
DDG AX + 25% glycerol	153.6	52.6 ± 1.43
DDG AX + 50% glycerol	143.4	58.0 ± 0.90
LSD (*p* < 0.05)		2.1
LSD (*p* < 0.01)		2.1

^a^ Carbon content was only measured a single time for use in the calculation of percent biodegradable material; ^b^ wheat bran arabinoxylan; ^c^ maize bran arabinoxylan; ^d^ dried distillers grain arabinoxylan.
